# Antimicrobial bathing of the critically ill for the prevention of healthcare-associated infection at a hospital in California

**DOI:** 10.1017/ash.2024.311

**Published:** 2024-09-16

**Authors:** Lilia Ryan, Brooke Livingston

**Affiliations:** John Muir Health System, Walnut Creek Medical Center, California; John Muir Health

## Abstract

**Background:** Patients in the intensive care unit (ICU) have invasive lines increasing their risk for healthcare-associated infections (HAIs). Our objective was to determine if antimicrobial bathing with 2% chlorhexidine gluconate (CHG) compared to the colloidal silver-based antimicrobial product would reduce the incidents of central line–associated bloodstream infections (CLABSI) and catheter-associated urinary tract infections (CAUTI). **Methods:** We performed a before-and-after study in four adult ICUs at a two-hospital facility in California. Prospective surveillance of CLABSI and CAUTI prevention bundles monitoring was established. The intervention consisted of daily bathing with CHG for all patients in the ICUs. A baseline period of one year was followed by an intervention period of one year. The incidence rates of CLABSIs and CAUTIs were compared between the baseline and intervention periods utilizing a t-test analysis. **Results:** A total of 10103 patients were included. At Facility A, a mean CLABSI rate of 2.43/1000 central line catheter days (CL) with 2149 patients days (Mean differences 95% CI -0.5–3.1; P>0.0975), during the baseline period followed by 1.11/1000 CL days with 2193 patient days in the intervention period. At Facility B, the mean CLABSI rate of 1.82/1000 CL days with 2976 patient days (Mean differences 95% CI -0.6–2.31; P>0.161) during the baseline period was followed by 1.01/1000 CL days with 2785 patient days in the intervention period. At Facility A, the mean CAUTI rate of 1.37/1000 indwelling catheter days (IUC) with 2149 ICU patient days (Mean difference 95% CI is 0.28–1.97; P 0.2160) was noted in the baseline period, followed by 0.45/1000 IUC days with 2785 in the intervention period. **Conclusion:** Daily bathing with CHG significantly reduced the incidence of CAUTI at Facility A. It is unclear why Facility A saw a statistically significant reduction in CAUTI, but Facility B did not. The difference in outcomes may be related to hospital size, service lines, supply constraints, and discrepancies in staffing. CHG bathing was not directly associated with a reduced risk of CLABSI at Facility A and B during our limited study, but it was encouraging enough that our organization will continue this intervention to obtain additional data to determine if bathing with CHG will reduce CLABSI and CAUTI.

